# Mortality from Pandemic Influenza A (H1N1) in Iran

**Published:** 2011-10-01

**Authors:** M M Gouya, M Nabavi, M Soroush, A A Haghdoust, S Ghalehee, P Hemmati, M Nasr Dadras, M K Fallahzadeh, K B Lankarani

**Affiliations:** 1 Center for Disease Control, Ministry of Health and Medical Education, Tehran, Iran; 2 Department of Epidemiology and Biostatistics, School of Health, Kerman University of Medical Sciences, Kerman, Iran; 3 Health Policy Research Center, Shiraz University of Medical Sciences, Shiraz, Iran

**Keywords:** Influenza A, H1N1, Mortality, Epidemiology, Iran

## Abstract

**Background:**

Due to worldwide spread of influenza A (H1N1) virus, the World Health Organization declared the first pandemic of influenza in four decades. This study aims to report the mortality from pandemic influenza A (H1N1) in Iran population and its epidemiologic and clinical characteristics up to December 21, 2009.

**Methods:**

The data were obtained from all provinces and reported to center for disease control of Ministry of Health and Medical Education (MOHME) of Iran through nationwide surveillance system for influenza A (H1N1) was implemented by MOHME since April 2009.

**Results:**

Of 3672 confirmed cases of influenza A (H1N1) in Iran between 22 May and 21 December 2009, 140 (3.8%) deaths were reported, mostly in 15-65 year old (yo) age group (67%). The highest admission mortality rate was in > 65 yo group (107 deaths/1000 hospitalized cases). Of decedent patients, 54% had no long term condition or risk factor, 34% had one, 11% had two, and 1% had three. Diabetes mellitus, pregnancy, chronic respiratory diseases and hypertension were the most common underlying conditions. The most common clinical pictures of death were acute respiratory distress syndrome and viral pneumonia. Although 66% of decedent patients received oseltamivir, enough information was not available about time of onset of antiviral therapy.

**Conclusion:**

As death due to influenza A (H1N1) occurs in all age groups and in those with and without any predisposing factors, we recommend health policy makers to provide influenza vaccination for people with underlying conditions and respiratory hygiene for all people.

## Introduction

Due to worldwide spread of influenza A (H1N1) virus, on June 11, 2009, the World Health Organization (WHO) formally declared the first pandemic of influenza in four decades.[1] Compared to seasonal flue, infection with influenza A (H1N1) usually results in a milder illness; however, a clinical spectrum comparable to seasonal influenza has been described.[2][3][4] Furthermore, the age groups affected by influenza A (H1N1) were reportedly younger.[5][6] Similar to the seasonal influenza, risk of fatality was reported to be higher in adults with 65 years of age or more, and those with certain chronic medical conditions.[5][7] Up to early June 2010, more than 18000 deaths attributable to influenza A (H1N1) have been reported worldwide.[4] So far, several studies have described mortalities associated with influenza A (H1N1) virus and its risk factors worldwide with various degrees of comprehensiveness. The aim of this study was to describe the data related to deaths due to influenza A (H1N1) infection including demographic characteristics of decedent patients, risk factors for death and use of antiviral treatment by these patients in Iran by December 2009.

## Materials and Methods

The data about decedent patients with influenza A (H1N1) in this article were obtained through a nationwide surveillance system for influenza A (H1N1) implemented by Ministry of Health and Medical Education (MOHME) of Iran since April 2009. This surveillance system was established in all provinces of Iran shortly after the announcement of first human cases of influenza A (H1N1) by WHO. It was based on multiple expert panels at the onset of the pandemic in Iran and also the ongoing experience with surveillance of avian flu (started since September 2005). The methods of sampling and data gathering by this surveillance system were previously described by Gooya et al.[8]

A confirmed case of influenza A (H1N1) was defined as a patient with high grade fever (>38°C) or at least two of following respiratory symptoms: cough sore throat, nasal obstruction/rhinorrhea, and fever/ feverishness; the patient should also have H1N1 viral infection confirmed by reverse transcriptase PCR (RT-PCR).

Influenza A (H1N1)-related death was defined as any patient with a confirmed influenza A (H1N1) infection in an ante mortem or post mortem specimen, who died from a clinically compatible illness or complications attributable to that infection, with no complete recovery between the onset of illness and demise and also no alternative cause of death.

## Results

**Fig. 1:**
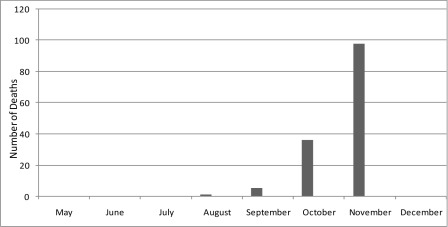
Monthly incidence of deaths among confirmed cases of pandemic A/H1N1 from May 22 to December 21, 2009 in Iran.

Out of 3672 confirmed cases of influenza A (H1N1) in Iran between 22nd May and 21st December 2009, 140 (3.8%) deaths (69 males and 71 females) were reported. The time distribution of these deaths was shown in Figure 1; most deaths occurred in October and November while no mortality was reported in May, June, July and December.

Decedent patients ranged in age from 0 to 95 years (median age=27.5 years). Decedent patients were divided into four age groups according to age classification of WHO. The number of deaths, case fatality rates and also admission mortality rates in these age groups were demonstrated in Table 1.

Of decedent patients, 75 (54%) had no long term underlying condition or risk factor, 47 (34%) had one, 15 (11%) had two, and 2 (1%) had three. There was not enough information about one patient. The frequency of long term conditions and risk factors in decedent patients were demonstrated in Table 2. Diabetes mellitus, pregnancy, chronic respiratory diseases and hypertension were the most common risk factors in decedent patients.

Clinical pictures of death in decedent patients were shown in Table 3; the most common picture was acute respiratory distress syndrome (ARDS) followed by viral pneumonia. Clinical picture of death was not reported in one patient.

Among decedent patients, 93 (66%) received oseltamivir; however, no information was available about the time between the onset of symptoms and initiation of oseltamivir therapy. Eleven (8%) patients initially presented to health care facilities with cardiopulmonary arrest. Eight decedent patients (6%) received no antiviral treatment. There was not sufficient information about the status of antiviral therapy in 28 (20%) patients.

**Table 1:** Age specific number of deaths and admission mortality rates among reported confirmed cases of Pandemic 2009 Influenza A (H1N1) - Iran, May 22 through December 21.

**Age group (years)**	**Number of deaths (%)**	**Admission mortality rate (deaths per 1000 hospitalized cases)**
<5	17 (12)	64
5-15	18 (13)	43
15-65	94 (67)	48
>65	11 (8)	107

**Table 2:** History of long term conditions and other risk factors among the patients dying from pandemic A/H1N1. Numbers in parentheses indicate number of patients with single long term condition or risk factor.

**Risk factor**	**No. (%)**
Respiratory:	
Asthma	4 (3)
COPD	8 (6)
Pulmonary Tuberculosis	1 (1)
Cardiac:	
Hypertension	9 (1)
Ischemic Cardiovascular Disease	1 (0)
Heart Failure	5 (1)
Other	1 (1)
Endocrine:	
Diabetes mellitus	13 (2)
Hematological:	
Fanconi anemia	1 (1)
Β-thalassemia major	2 (1)
Malignancy	5 (5)
Other	1 (1)
Neurological:	
Cerebral palsy	3 (3)
Down syndrome	5 (4)
Epilepsy	2 (2)
Stroke	1 (0)
Renal:	
Chronic kidney disease	5 (2)
Gastrointestinal:	
Celiac Disease	1 (1)
Rheumatologic Disease	1 (1)
Pregnancy	11 (10)
Drugs:	
Chronic corticosteroid consumption	2 (0)
Addiction	1 (1)

**Table 3:** Clinical pictures of death among the patients dying from pandemic A/H1N1. Numbers in parentheses indicate number of patients with single clinical picture of death.

**Disease**	**No. (%)**
Respiratory	
Acute respiratory death syndrome (ARDS)	81 (77)
Viral pneumonia	29 (27)
Secondary bacterial pneumonia	3 (3)
Cardiovascular	
Myocarditis	7 (6)
Myocardial infarction	1 (1)
Cardiac Arrest	1 (1)
Pulmonary emboli	1 (1)
Neurologic	
Meningoencephalitis	12 (11)
Sinus vein thrombosis	1 (0)
Status epilepticus	1 (0)
Renal	
Acute Tubular Necrosis	2 (0)
Homeostatic	
Internal hemorrhage due to bleeding tendency	4 (3)
DIC	1(1)

## Discussion

Between 22nd May to 21st December 2009, 140 confirmed deaths due to influenza A (H1N1) were reported in Iran. Most deaths occurred in October and November with no mortality was reported in May, June, July and December. A large proportion of decedent patients had no predisposing risk factor or underlying condition. This is in contrast with reports from other countries where majority of patients had predisposing risk factors and underlying conditions.[5][7][9][10]

However, underlying conditions like diabetes mellitus, hypertension, chronic respiratory diseases like asthma and COPD, and pregnancy which were common predisposing factors in Iranian population, were also reported to be common underlying conditions in decedent patients in other reports.5,7,9,10 While most deaths occurred in those aging 5 to 65 years, the highest admission mortality rates were observed in those aging more than 65 years. In a crosssectional study by Moghadami et al.,[11] it has been demonstrated that a large proportion of Iranian people were seropositive for polyclonal antibody against influenza A (H1N1); no difference was noted between those with and without history of flu-like illness in past six months. The authors proposed that this lack of difference could be explained by high rates of asymptomatic infection with influenza A (H1N1). Hence, it is very hard to estimate the exact number of Iranian patients infected with influenza A (H1N1) during the period of study. Therefore, we preferred to use admission mortality rate instead of case fatality rate in this study. Similar to our findings which showed a J shaped age specific admission mortality rate, other studies have reported similar figures with highest case fatality rates in those aging more than 65 years.[5][7][12]

Although 66% of decedent patients received antiviral treatment, no information was available about its timing. Taking antiviral treatments, particularly in the first 48 hours of illness, has been reported to confer a survival benefit to the patients;[13] therefore, it is probable that most of decedent patients in our study who used antiviral drugs did not receive this treatment in the first two days after onset illness.

## Conclusion

Influenza A (H1N1) resulted in 140 confirmed deaths in Iranian population. Most deaths occurred in patients aging 5 to 65 years; however, the highest admission mortality rate was observed in those older than 65 years. Although majority of the decedent patients had no risk factor or underlying condition, diabetes mellitus, pregnancy, hypertensions and chronic respiratory diseases were among the most common reported conditions in decedent patients.
